# Generative Adversarial Learning Enhanced Fault Diagnosis for Planetary Gearbox under Varying Working Conditions

**DOI:** 10.3390/s20061685

**Published:** 2020-03-18

**Authors:** Weigang Wen, Yihao Bai, Weidong Cheng

**Affiliations:** School of Mechanical, Electronic and Control Engineering, Beijing Jiaotong University, Beijing 100044, China; 18121250@bjtu.edu.cn (Y.B.);

**Keywords:** planetary gearbox, cross-domain, intelligent fault diagnosis, generative adversarial learning, varying working conditions

## Abstract

Planetary gearbox is a critical component for many mechanical systems. It is essential to monitor the planetary gearbox health and performance in order to maintain the whole machine works well. The methodology of mechanical fault diagnosis is increasingly intelligent with the extensive application of deep learning. However, the cross-domain issue caused by varying working conditions becomes an enormous encumbrance to fault diagnosis based on deep learning. In this paper, in order to fully excavate potentialities of deep neural network architectures, a novel generative adversarial learning method was introduced for a completely new fault diagnosis based on a deep convolution neural network. In addition, the intelligent fault diagnostic scheme for planetary gearbox under varying speed conditions was developed. After that, some experiments on measured vibration signals of planetary gearbox were conducted to verify the validity and efficiency of the fault diagnostic scheme. The results showed that the proposed method enhanced the capability of the intelligent diagnosis for planetary gear faults under varying speed conditions.

## 1. Introduction

Planetary Gearbox (PG) is widely used in different sorts of machinery, owing to its compact structure, large transmission ratio, strong load capacity, and high efficiency, such as helicopters, wind turbines and robots in manufacturing systems. The vital role that the planetary gearbox plays in a mechanical system makes its fault diagnosis become really significant. Most researchers use the vibration signal of the machine to diagnose its health status. However, the information inside the vibration signal is intricate and masked by many noise components. Lei and Feng built mathematical vibration models to show the multi-frequency components inside the signal and their experiments validated the fact that the planetary gearbox vibration signal was complicated for fault diagnosis [1,2]. So, from the vibration mechanism, fault feature extraction, to fault pattern recognition, these research topics on fault diagnosis via vibration signal have been widely studied in the field of planetary gearbox fault diagnosis [3].

While various methods have been developed for the fault diagnosis of planetary gearboxes, the intelligent fault diagnosis method is more and more widely utilized to tackle complicated mechanical diagnosis problems due to its adaptive learning mechanism, strong fault tolerance and high non-linear regression ability [4,5]. Combining signal processing methods and machine learning algorithms, researchers have proposed many intelligent fault diagnosis schemes for planetary gearbox. Cheng and Chen proposed a PG diagnosis scheme which combined the fused entropy feature from Ensemble Empirical Mode Decomposition (EEMD), machine learning algorithms of Kernel Principle Component Analysis (KPCA) and Learning Vector Quantization (LVQ) [6]. Li used the signal processing method based on Adaptive Multi-scale Morphological Filter (AMMF) and Modified Hierarchical Permutation Entropy (MHPE) to extract fault features in PG vibration signals and then diagnosed the fault by Binary Tree Support Vector Machine (BT-SVM) [7]. Lei also advanced a health condition identification method for multi-stage PG, adopting multi-class relevant vector machine as a classifier and introduced Accumulative Amplitudes of Carrier Orders (AACO) and Energy Ratio-based Difference Spectra (ERDS) as fault features which improved diagnosis performance and robustness [8]. These intelligent methods had certain limitations and uncertainties. Firstly, in the pre-processing stage of vibration signal, the expertise knowledge was highly required, such as signal processing and data statistics. On the other hand, the intrinsic features for the faults of planetary gearbox were not found in these existing literatures, which were independent of the fluctuation of working conditions. Thus, these led to the problem of cross-domain for intelligent fault diagnosis of PG.

In recent years, Deep Learning (DL) has been attracting growing attention from various fields. The deep model hierarchical structures make DL capable of learning intrinsic representations of raw complex data, which provide the foundation for its popular application in visual recognition and natural language processing [9,10,11,12,13]. Researchers in the field of mechanical fault diagnosis have taken advantage of the deep learning ability to realize more adaptive feature learning from vibration signals [14]. Some researchers applied various sorts of deep learning models as an upgraded classifier with manually extracted features [15,16,17,18,19,20,21,22,23,24,25,26,27,28]. Such as Li and Sanchez utilized a deep support vector classification to diagnose gearboxes and bearings with statistic features in time, frequency and time-frequency domains [21]; Chen and Li extracted several time and frequency features and employed a Deep Belief Network (DBN) to classify different health status of a gearbox [22]; and Shao and Jiang composed an optimized DBN with 18 time-domain features of signals as an input to enhance fault diagnosis of bearings [28]. Among these methods, the fault features had to be extracted firstly by complex signal processing methods, and DL models were only used as a replacement of traditional machine learning algorithms. Beyond 2014, some researchers began to apply DL models on mechanical fault feature extraction. Lei and Jia proposed a Deep Auto Encoder (DAE)-based scheme to diagnose the health status of the multi-stage gearbox in the frequency domain, which reached 100% accuracy under a single working condition [29]. Janssens and Slavkovikj adopted a convolutional neural network to autonomously learn useful features for bearing fault detection in a frequency domain which performed better than using another classifier with manually chosen features [30]. Jing and Zhao utilized a convolution neural network to diagnose seven kinds of gearbox faults in four kinds of working loads, achieving 83% testing accuracy in the time domain and 98% accuracy in the frequency domain [31]. Zhang and Peng proposed a Deep Convolutional Neural Networks with a Wide first-layer kernels (WDCNN) model to diagnose bearing faults in the untrained working speeds data domain, which achieved nearly 100% accuracy, and preliminary showed the strong feature extraction ability of DCNN in mechanical fault diagnosis [32]. Many researchers also improved the CNN architecture to achieve better results for mechanical fault diagnosis [33,34,35]. However, few of them considered the issues of the cross domain caused by the varying working conditions and environments. So, recently, researchers introduced other deep learning methods, such as transfer learning, to address the cross-domain problem [36,37].

As for the DL-based fault diagnoses, they have tremendous ability of feature extraction and state recognition in different domains if there are sufficient various data for training. However, it is a notoriously slippery task to get enough labeled data in various domains for diagnosis model training to overcome the problem of cross-domain, especially in the practical engineering field. In this paper, Generative Adversarial Network (GAN) is introduced for training the fault diagnosis model to fully exploit the potentialities of the intelligent diagnostic scheme without enough experimental data. The GAN-enhanced CNN is constructed for intelligent fault diagnosis of PG. Through theoretical design and experimental verification, the methodology is better for the PG fault diagnosis under varying working conditions than normal CNN. 

The rest of the paper is organized as follows. The generative adversarial learning architecture is described in Section 2. The intelligent fault diagnosis method based on GAN-enhanced CNN is presented in Section 3. Section 4 details the experiment validation for the method. Section 5 concludes the paper at last.

## 2. Generative Adversarial Learning Architecture

GAN framework is introduced for intelligent fault diagnosis model training, which consists of two neural networks trained in opposition to one another. The generator *G* takes a random noise vector *z* as an input and outputs a series of signal *X_fake_* = *G*(*z*), which simulates the vibration signal of PG. The discriminator *D* receives either a real vibration signal or a generated fake signal from the generator as input and outputs of a probability distribution *P*(*S* | *X*) = *D*(*X*) over possible signal sources. The discriminator is trained to minimize the log-likelihood loss function *Ls* that forces it to assign to the correct source:

*Ls* = − *E*[log *P*(*S* = *real* | *X_real_*)] − *E*[log *P*(*S* = *fake* | *X_fake_*)] (1)

**Figure 1:**
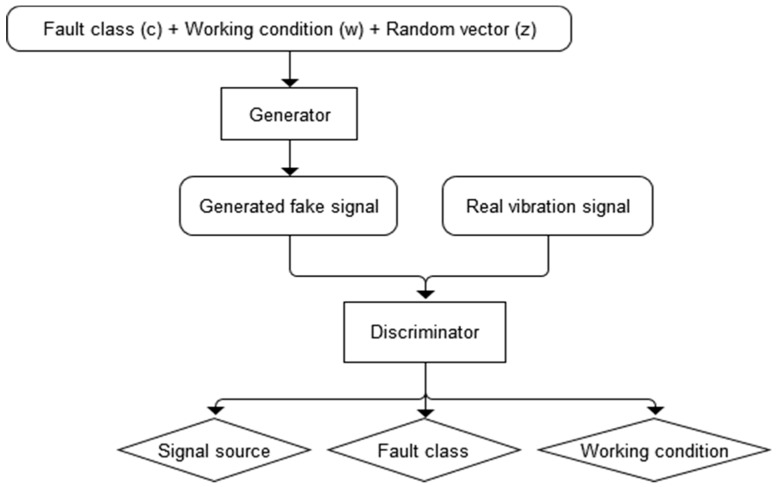
Generative adversarial learning architecture.

In addition, the generator in GAN is trained to minimize the quadratic term in Equation (1). However, the fault status and the working conditions have important influences in the formation of PG vibration signals. The fault class and working condition are employed as inputs independent of *z*. The Auxiliary Classifier GAN (ACGAN) framework is adopted as shown in Figure 1. 

Every generated sample has a corresponding fault class label, *c* ∼ *p_c_* and working condition label, *w* ∼ *p_w_* in addition to the noise *z*. *G* uses all of these to generate vibration signals *X_fake_* = *G*(*c, w; z*). The discriminator gives both a probability distribution over sources and a probability distribution over the labels, such as *P*(*S* | *X*), *P*(*C* | *X*) and *P*(*W* | *X*). The loss function has three parts: the log-likelihood of the correct source, *L_S_* of Equation (1); the log-likelihood of the correct fault class, *L_C_* of Equation (2); and the log-likelihood of working condition, *L_W_* of Equation (3).

*L_C_* = − *E*[*log P*(*C* = *c* | *X_real_*)] − *E*[*log P*(*C* = *c* | *X_fake_*)] (2)

*L_W_* = − *E*[*log P*(*W* = *w* | *X_real_*)] − *E*[*log P*(*W* = *w* | *X_fake_*)] (3)

In the ACGAN scheme, *D* is trained to minimize *L_S_* + *L_C_* + *L_W_* while *G* is trained to minimize *L_C_* + *L_W_* − *L_S_*. This ACGAN frameworks can learn a representation for *z* that is independent of the fault class label and working condition.

Mode collapse is a vital issue to consider when training and implementing the GAN framework. The training is said to result in mode collapse if the generator ends up mapping multiple *z* vectors to the same output *x*, which is assigned a high probability of being real by the discriminator. The common view of mode collapse and instability in GAN training is that it is caused by the supports of real and model distributions being disjoint or lying on low-dimensional manifolds, and the generator cannot get useful gradients to learn [38]. A novel gradient penalty scheme called Deep Regret Analytic Generative Adversarial Networks (DRAGAN), which enables faster training, achieves improved stability and modeling performance is applied here. The strategy to mitigate mode collapse is to regularize the discriminator using the following penalty [39].

*L_R_* =*E_x∼Preal,δ∼Nd_*_(0,*cI*)_[|| ∇*_x_ D*(*x* + *δ*) || − *k*]^2^ (4)

where ∇*_x_* is the gradient against real data *x*. This works as a small perturbation of real data, which is to constrain its gradients in the ambient data space and is likely to lie off the data-manifold. In the training process, the instance noise *δ* of *N_d_*(0,*cI*) is input to the discriminator with real data. The loss functions for discriminator *D* and generator *G* can be synthesized as

*L*(*D*) = *λ_S_L_S_* + *λ_C_L_C_* + *λ_W_L_W_* + *λ_R_L_R_* (5)

*L*(*G*) = *λ_C_L_C_* + *λ_W_L_W_* − *λ_S_L_S_* (6)

where *L*(*D*) is the loss function of the discriminator, and *L*(*G*) is the loss function of the generator; *λ_S_*, *λ_C_*, *λ_W_*, and *λ_R_* are the coefficients of each loss in the synthesized loss function, which are selected as 1, 3, 5, and 0.5 in the later experiments.

## 3. Fault Diagnosis Method Based on GAN Enhanced CNN

### 3.1. One-Dimensional Convolution of CNN

A deep CNN architecture is always composed of several convolution blocks to enhance the feature representation ability of CNN. A convolution block is normally composed of convolution layers, activation layers, pooling layers, and so on. According to the extracted features by convolution block, some activated fully connected layers and soft-max function follow afterward, acting as a classifier. The convolution layer performs the convolution operation on the input signal to produce an output to the next layer. Because the vibration signal is a one-dimensional time series, the convolution kernel vector with size $K_{j}\times 1$ is applied here; the output of the convolution operation is:

(7) $$y_{j}=xw_{j}$$

(8) $$y_{j}\left[n\right]=\sum_{k=1}^{K_{j}}x\left[n-k+1\right]w_{j}\left[k\right]$$

where $y_{j}$ is the output of convolution, x is input data, and $w_{j}$ is the jth kernel. Formula (7) is the definition, and (8) is the calculation of the convolution. Since x is the vibration signals or mapped features defined in the time domain, the convolution operation corresponds to a Finite Impulse Response (FIR) filtering operation, and the convolution kernel can be regarded as a passband FIR filter. According to this band-pass filtering property of the convolution kernel, each convolution operation can extract features within a special frequency band, and the output of all convolution operations in a convolution layer can be accumulated. So, the output of the convolution layer is

(9) $$Y\left[n\right]=\sum_{j}\sum_{k=1}^{K_{j}}x\left[n-k+1\right]w_{j}\left[k\right]$$

Because the bias effect will be counteracted in the next batch normalization layer, the bias parameters in the convolution formula are omitted. By mean of accumulation and abbreviation, the characteristics in multiple frequency bands can be represented in only one feature map, so the number of network parameters is reduced dramatically and the CNN architecture can be made deeper.

### 3.2. Batch Normalization

The vibration signal of PG is unstable because of the variable working conditions. Batch Normalization (BN) is designed to re-scale the input feature map to reduce the shift of internal covariance. The transformation of the BN layer is

(10) $$BN=\gamma \frac{x-E\left[x\right]}{\sqrt{V\left[x\right]+\epsilon }}+\beta$$

where E[x] and V[x] are the expectation and variance of the corresponding input feature maps; ϵ is a very small constant in case of dividing zero; and γ and β are the scale and shift parameters, which are trainable. This operation standardizes the input feature and learns the covariance shift between different batches of input data to adjust the output features, which significantly solves the problems of gradient vanishing and gradient exploding. It converts the original unknown data distribution into a new fixed distribution, which fits right the feature that the model learns.

### 3.3. Rectified Linear Units Activation

For increasing the nonlinear properties of the final decision function without affecting the receptive fields of the convolution layer, nonlinear activation function *f* is adopted after the convolution layer. The activation output is defined as

(11) $$y^{\prime }=f\left(y\left(i\right)\right)$$

Rectified Linear Units (ReLU) is often preferred to the other activation functions in CNN. ReLU activation function applies the non-saturating activation function to the input features. The employed ReLU activation function is

(12) f(y(i))=max{0,y(i)}

where y(i) is the input feature map. ReLU effectively decreases the parameter mutual dependency and reduces the chance of the vanishing gradient problem in the deep network because its gradient is either 0 or 1. It will increase the feature sparsity and improve general adaptive capability of CNN.

### 3.4. Generator of Vibration Signal

A 128-dimensional random noise vector *z* is taken as the latent space for the generator. The side information includes fault class and working condition information. The fault class is one-hot encoded into a 64 × 1-dimensional vector *c*. Here, the rotation speed working condition is just considered according to the real data samples in our experiments. Binary encoding is used to encode the attribute of rotation speed as a 64 × 1-dimensional vector *w*. The vector *z*, *c* and *w* are connected together into a 256 × 1-dimensional vector, which is input into the generator. The input vector is extended into 512 dimensions through the fully connected layer at first. Then, 10 convolution blocks are utilized to modulate the input signal into different pattern signals. After that, four deconvolution blocks are used to up-sample the adequately modulated signal into a 8192 × 1-dimensional fake signal.

**Figure 2:**
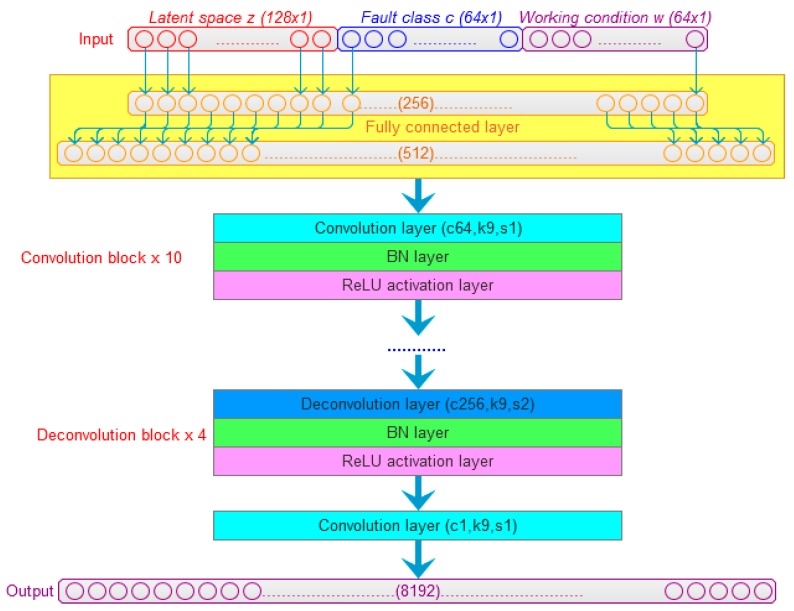
The generator of vibration signal.

As shown in Figure 2, the convolution block is composed of a convolution layer, a BN layer and a ReLU activation layer. Here, the 1-D convolution kernel is used. In the convolution layers, “c64, k9, s1” means that there are 64 convolution kernels; the 1-D kernel size is 9 × 1; and the stride size is 1. The deconvolution block is composed of a deconvolution layer, a BN layer and a ReLU activation layer, and “c256, k9, s2” means that there are 256 deconvolution kernels, the 1-D kernel size is 9 × 1, and the stride size is 2. The last convolution layer amplifies and accumulates the signals of each channel, and outputs the final synthetic signal.

### 3.5. Discriminator of Fault Diagnosis

**Figure 3:**
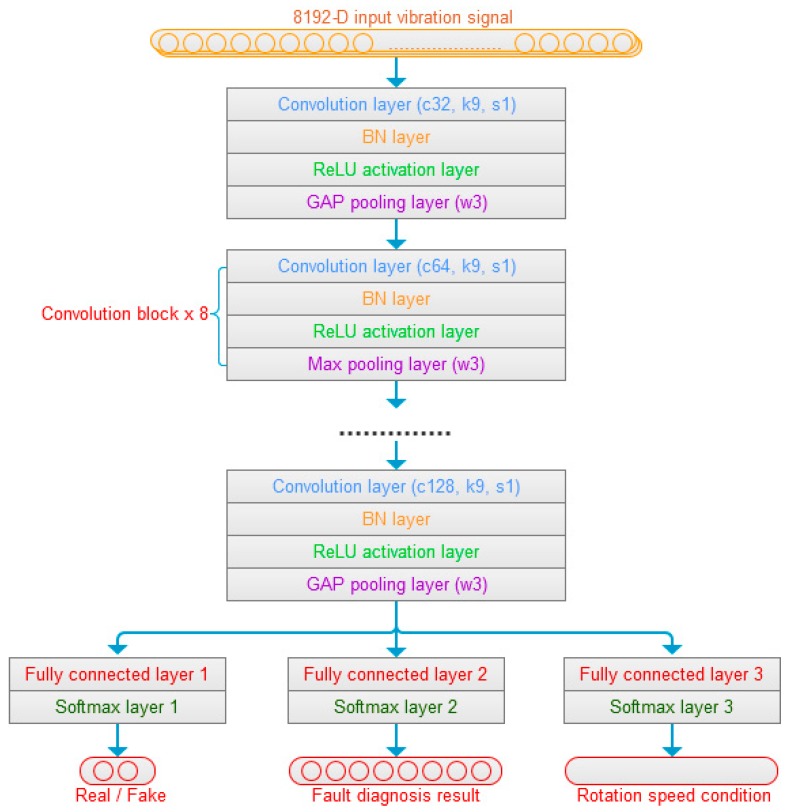
The discriminator construction.

The PG vibration signals or fake signals are input to the discriminator of fault diagnosis. As shown in Figure 3, the first convolution block of the discriminator is composed of a convolution layer, a BN layer, a ReLU activation layer, and a Global Average Pooling (GAP) layer. “c32, k9, s1” of the convolution layer means that there are 32 convolution kernels, the 1-D kernel size is 9, and the stride size is 1 in the convolution layer. Here, the GAP pooling layer acts as an anti-aliasing low-pass filter to reduce the noise effect, and “w3” in pooling layer means the pooling size is 3, which corresponds to different cut-off frequencies. The next eight convolution blocks are composed of a convolution layer, a BN layer, a ReLU activation layer, and a max pooling layer, which extract multi-scale features of signal. The three fully connection layers and soft-max layers are adopted to realize the recognition of signal sources, classification of the faults, and regression of rotation speed conditions at last.

## 4. Experiment Validation

**Figure 4:**
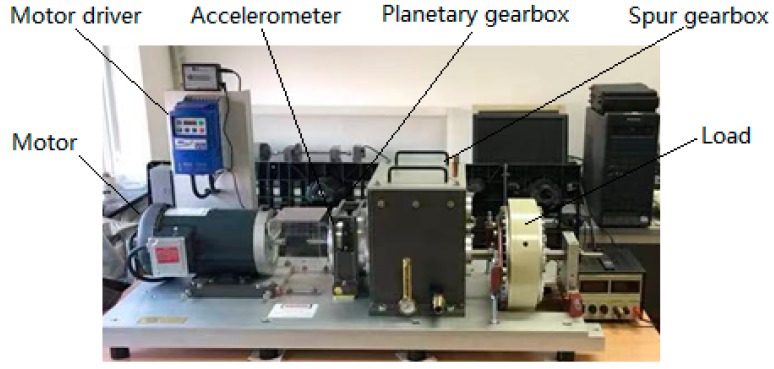
The planetary gearbox fault diagnosis test rig.

**Figure 5:**
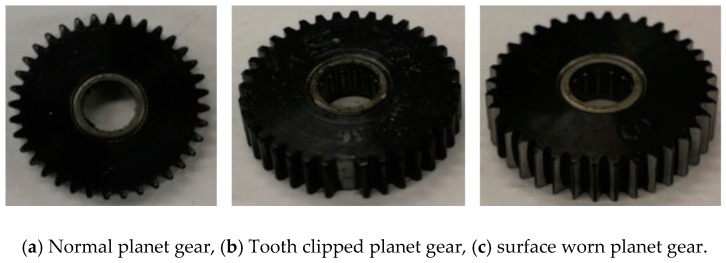
Different health states of planet gear.

The vibration signals for validation of GAN-enhanced CNN were collected from the planetary gearbox fault diagnosis test rig shown in Figure 4. The motor drives the input shaft of the planetary gearbox, which is fixed to the sun gear. The ring gear is standstill, and the planet carrier is coupled to the output shaft, which drives the spur gearbox and the load. The rotation speed of the planetary gearbox input shaft is measured by the tachometer. The vibration signals are sampled by an accelerometer mounted on the planetary gearbox. The experimental planet gears are shown in Figure 5. As shown in the figure, three kinds of PG health states are made, which are normal planet gear(NA) in (a), man-made tooth clipped planet gear(TC) in (b), and teeth surface worn planet(SW) gear in (c).

The raw vibration signals of the planetary gearbox were sampled with a sampling frequency of 12,000 Hz. For three health states of a normal gearbox, gearbox with a tooth clipped planet gear, and gearbox with a surface worn planet gear, the vibration signals were collected under five speed conditions, and in total, 15 segments of vibration signals were obtained. About 10,000 pieces of data sets with 8192 data points were made in each segment through sliding window. The working frequency details of the vibration signals are shown in Table 1. The Working Condition A (WC-A) is an input rotation speed of 1500 r/min, the Working Condition B (WC-B) is an input rotation speed of 1200 r/min, the Working Condition C (WC-C) is an input rotation speed of 900 r/min, the Working Condition D (WC-D) is input rotation speed of 600 r/min, and the Working Condition E (WC-E) is an input rotation speed of 300 r/min. 

### 4.1. Experiment of Vibration Signal Generation

**Figure 6:**
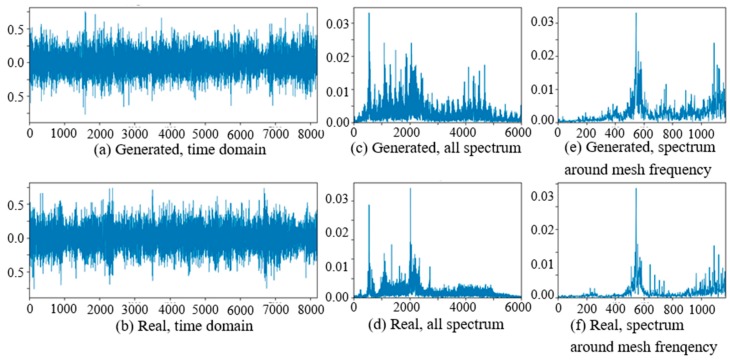
Comparison of normal planetary gearbox vibration signal under WC-A.

**Figure 7:**
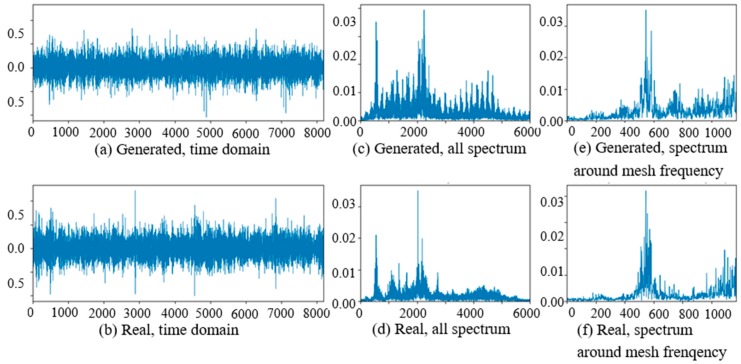
Comparison of planetary gearbox with clipped planet gear vibration signal under WC-A.

**Figure 8:**
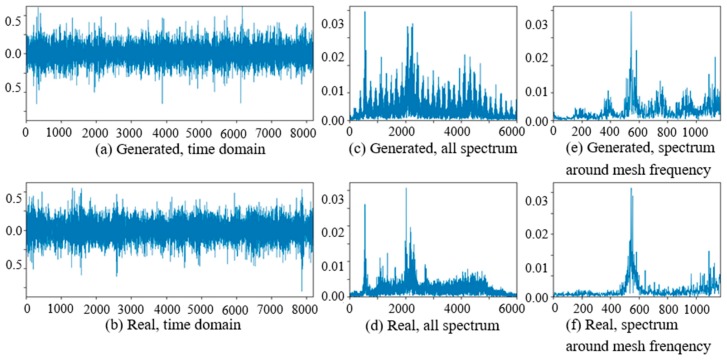
Comparison of planetary gearbox with worn planet gear vibration signal under WC-A.

**Figure 9:**
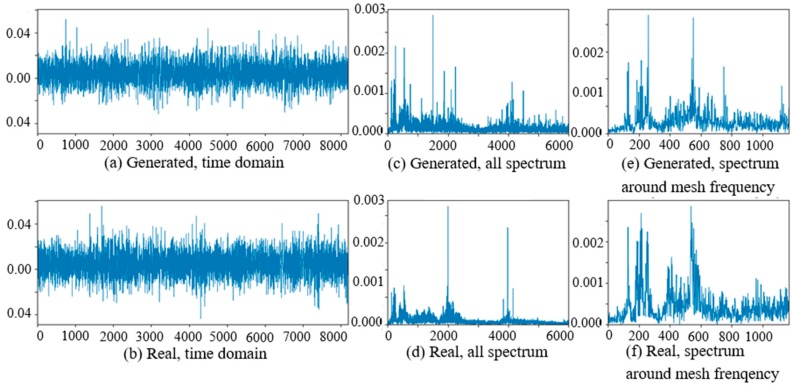
Comparison of normal planetary gearbox vibration signal under WC-E.

**Figure 10:**
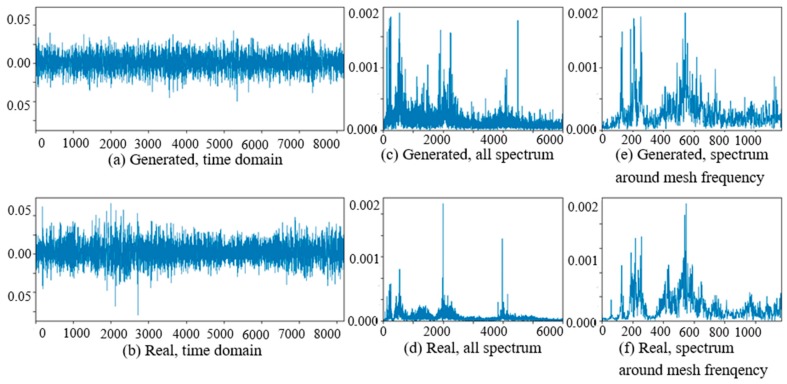
Comparison of planetary gearbox with clipped planet gear vibration signal under WC-E.

**Figure 11:**
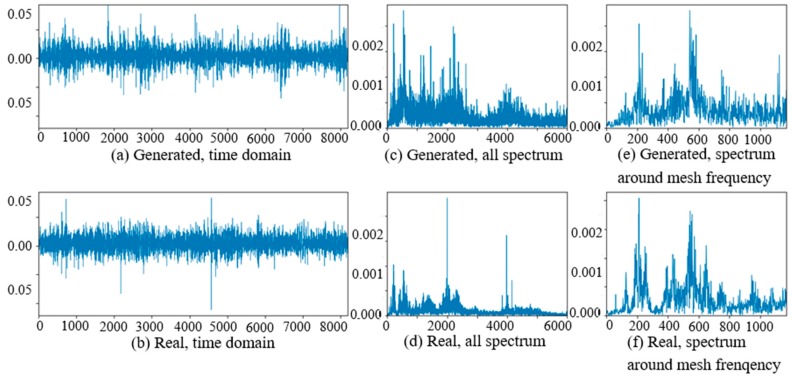
Comparison of planetary gearbox with worn planet gear vibration signal under WC-E.

At the beginning of the experiment of PG vibration signal generation, the WC-D were treated as the target domain for the uncertain various speed conditions, and the rest of the four kinds of test data sets (WC-A, WC-B, WC-C, WC-E) composed the source domain data as the training data sets. After the training of GAN-enhanced CNN with the test data sets in the source domain, the generator could generate the vibration signals of normal gearbox, gearbox with tooth clipped planet gear, and gearbox with surface worn planet gear under various rotation speed conditions. The comparisons of real vibration signals and generated vibration signals of different state gearboxes in source domains were made at first. For representation of generated vibration signals and real vibration signals in source domains, Figure 6 to 11 show the time domain waveforms, all spectrums and the spectrums around the planet gear mesh frequency of the six vibration signals for the three health states (NA, TC, SW) under working conditions of WC-A and WC-E.

In Figure 6, Figure 7, Figure 8, Figure 9, Figure 10 and Figure 11, (a) and (b) are the time domain waveforms, (c) and (d) are the all spectrums, and (e) and (f) are the spectrums around the planet gear mesh frequency of the generated vibration signal and the real vibration signal. According to the comparison of information in three health states under WC-A and WC-E conditions, the time domain waveforms of the generated signals (a) and real signals (b) show the similar amplitudes and shapes, and there are almost the same frequency peaks and energy distributions in all the spectrums of (c) and (d), but the side bands around the planet gear mesh frequency are a bit different in the spectrums around the planet gear mesh frequency of (e) and (f). The energies of the generated signals disperse to more frequency bands than the real signals in the whole spectrums. The spectrums of the generated signal and real signal are more similar in Figure 9, Figure 10 and Figure 11 than in Figure 6, Figure 7 and Figure 8 because the signal-to-noise ratio of real signal under WC-E is lower than that under the higher speed condition of WC-A. So, the vibration signals generated by GAN can be regarded as the real one with more noise, and it is better for training of the CNN model.

**Figure 12:**
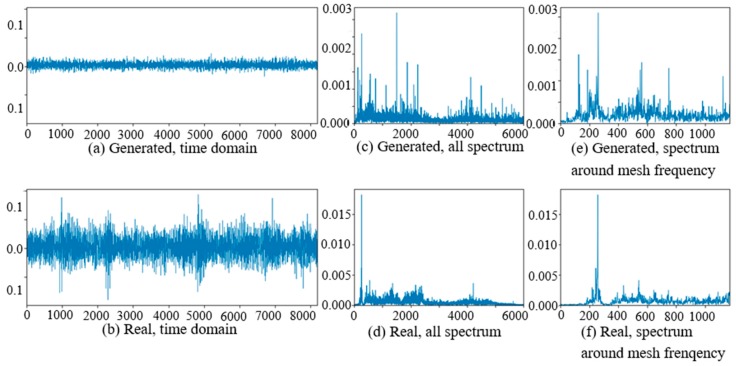
Comparison of normal planetary gearbox vibration signal in target domain.

**Figure 13:**
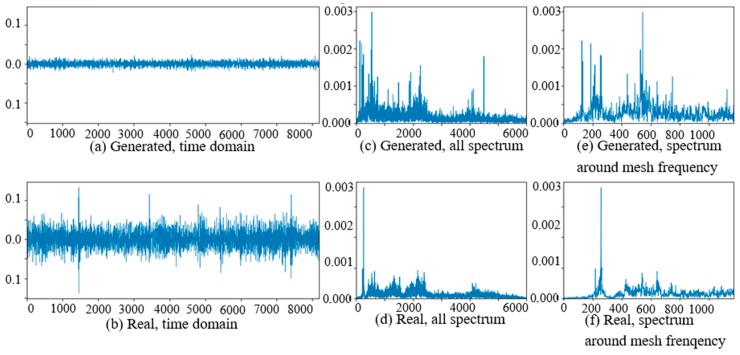
Comparison of planetary gearbox with clipped planet gear vibration signal in target domain.

**Figure 14:**
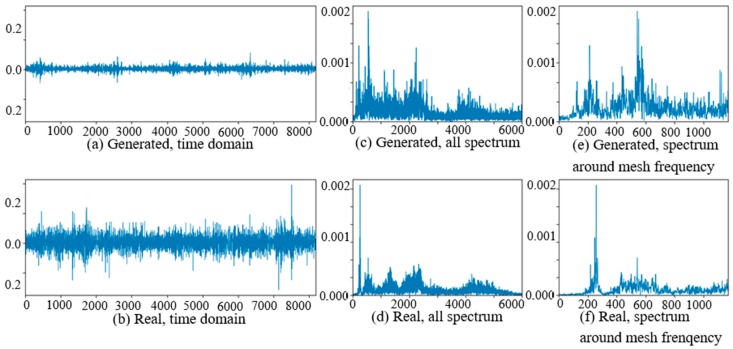
Comparison of planetary gearbox with worn planet gear vibration signal in target domain.

The comparisons of real vibration signals and generated vibration signals of different state gearboxes in the target domain of WC-D are shown as Figure 12, Figure 13 and Figure 14. In the figures, the amplitudes of the (a) generated signal time domain waveforms are smaller than (b) the real signals, but the waveform shapes are similar. On the same position of the low-frequency band in (c) and (d), there are corresponding prominent frequency peaks while there are many more frequency peaks and side bands around the planet gear mesh frequency in (e) the generated signal spectrum than (f) in the real signal spectrum. From the perspective of the all spectrum, there are more high-frequency peaks in the generated normal signal spectrums, while the spectrum forms of the generated fault signal and the real fault signal are similar in the middle and high frequency bands.

After training of GAN-enhanced CNN with the test data sets in source domain, not only can the generator produce the vibration signals, but also the discriminator can classify the fault of PG. In the experiments, the fault classification accuracy of a batch of generated vibration signals was applied as evaluation criterion. Table 2 shows the fault classification accuracy of the generated signals in the source domain and target domain. The fault classification accuracy of the generated signal in the source domain is in the interval of 83%–95% which is 78.7% in target domain. It means that all the generated signals have the same fault characteristics as the real vibration signals. These evidences prove the generator and the generated signals are effective measures for improving fault diagnosis ability of CNN.

### 4.2. Experiment of Fault Diagnosis in Target Domain

**Figure 15:**
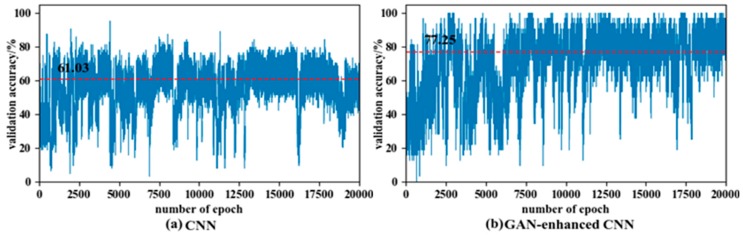
Comparison of cross-domain fault diagnosis performance of CNN and GAN-enhanced CNN.

In the experiment of the fault diagnosis in the target domain, the train sets are used to train the neural network model, and validation sets are used to evaluate the training quality. In verification of the GAN-enhanced CNN fault diagnosis ability in the target domain under various speed conditions, the four kinds of working conditions from the total five conditions were selected as source domains, and the other one was treated as the target domain. For comparison, at first the experimental data sets in the source domains of WC-A, WC-B, WC-C, and WC-E were utilized as training data for the CNN, and then the generated data in all the domains and the experimental data in source domains were employed for training the GAN-enhanced CNN. In order to reveal the detail of the differences between the CNN and the GAN-enhanced CNN, 100 data sets in the target domain of WC-D were tested at the end of each training epoch that was up to 20,000. The comparison of cross-domain fault diagnosis performance of CNN and GAN-enhanced CNN is shown in Figure 15. The blue curved line is the validation accuracy in the target domain after each training epoch, and the red dotted line is the average accuracy throughout the process. The validation accuracy in the target domain is improved by the GAN-enhanced CNN, and the average accuracy is increased from 61.3% to 77.25% in the figure. It demonstrates that the cross domain diagnostic capability of CNN is enhanced by the generated signals of the generator. 

**Figure 16:**
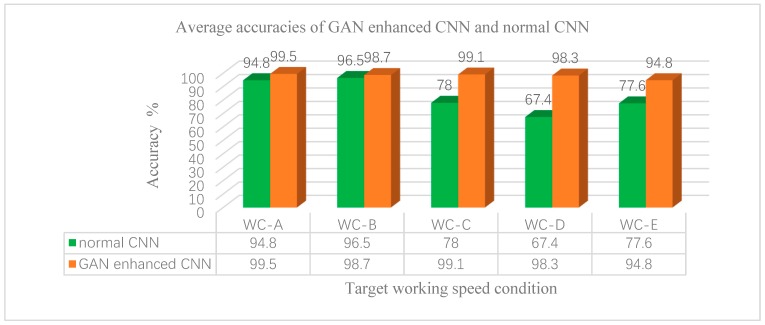
Average accuracies of GAN-enhanced CNN and normal CNN under various target conditions.

Then the other experiment was conducted in order to validate further how the GAN enhanced CNN model performed in different target domains under various speed conditions. When the neural network model has been trained to have satisfying performance on validation sets, it can be used in the diagnosing stage. The data sets in four kinds of speed conditions from the total five conditions were chosen as training data, and the data sets in the other one condition were employed as the target domain data sets that were used in the diagnosing stage. The result accuracies of GAN-enhanced CNN and normal CNN in the target domain are shown in Figure 16. We can see that the accuracies of the GAN-enhanced CNN are almost higher than normal CNN in all five kinds of target domains, and the highest accuracy reaches 99.5% in working condition WC-A, and the lowest accuracy reaches 94.8% in working condition WC-E. The normal CNN performs well in working condition WC-A and WC-B, which is only about 2–5% lower than that of GAN-enhanced CNN. However, the performance of normal CNN is pretty bad when the target domains are changed to working conditions of WC-C, WC-D and WC-E. It is because the fault characteristics are different in the vibration signal in the lower working speed, the normal CNN fails to extract the fault patterns under these kinds of working conditions, but generated signals of GAN carry the fault features in target domains for training. So, the GAN-enhanced CNN model has more powerful fault diagnosis capability in target domains under various working conditions.

## 5. Conclusions

The varying working conditions cause feature distribution changing of the vibration signal in the time domain and frequency domain, which will provoke the trouble of the cross-domain for intelligent fault diagnosis. The fault diagnosis of PG under varying working conditions is hard and tedious. We proposed an intelligent diagnostic scheme based on GAN-enhanced CNN to address the problem. The GAN architecture is designed combining the fault classes and working conditions of PG. The deep neural network model of the generator can simulate the dynamic model of PG and can generate the vibration signals of PG in different fault states under varying working conditions after generative adversarial learning. In addition, the CNN model of the discriminator is trained by the measured vibration signals in the source domain and enhanced by the generated vibration signals of the generator in the target domain. The cross-domain fault diagnosis accuracy of GAN-enhanced CNN under varying working conditions is as much as 99.5% in the experiments. The results prove that the cross-domain accuracy of GAN-enhanced CNN is 15.22% on average, and up to 30.9% higher than that of the normal CNN in the experiments. It is verified that the GAN-enhanced CNN can be utilized to diagnose the fault states of PG under varying working conditions with much accuracy. We will keep researching the fault diagnostic scheme that can measure the deviation between source domain and target domain and cross-domain intelligent fault diagnosis with only a few labeled training examples.

## Figures and Tables

**Table 1:** Planetary gearbox working parameters.

Parameter	Frequency (Hz)				
	WC-A	WC-B	WC-C	WC-D	WC-E
Sun gear rotation frequency	25	20	15	10	5
Planet gear meshing frequency	546.88	437.47	328.1	218.74	109.37
Planet carrier rotation frequency	5.47	4.38	3.28	2.19	1.09
Planet gear pass frequency	21.88	17.50	13.13	8.75	4.38
Planet gear rotation frequency	9.72	7.78	5.83	3.89	1.94
Faulty planet gear frequency	15.19	12.15	9.11	6.08	3.04

**Table 2:** The fault classification accuracy of the generated signals in source domain and target domain.

	Source Domain				Target Domain
	WC-A	WC-B	WC-C	WC-E	WC-D
classification accuracy	83%	88.3%	85.3%	95%	78.7%
